# Multimodal Detection of Magnetically and Fluorescently Dual-Labeled Murine Macrophages After Intravenous Administration

**DOI:** 10.3390/molecules30183726

**Published:** 2025-09-12

**Authors:** Anna N. Gabashvili, Sergey L. Znoyko, Anastasia V. Ryabova, Elizaveta N. Mochalova, Olga Yu. Griaznova, Tatiana A. Tortunova, Olga N. Sheveleva, Nina N. Butorina, Valeriia I. Kuziaeva, Irina V. Lyadova, Petr I. Nikitin

**Affiliations:** 1 Prokhorov General Physics Institute of the Russian Academy of Sciences, 119991 Moscow, Russia; gabashvili_a@nsc.gpi.ru (A.N.G.); znoykos@yandex.ru (S.L.Z.); nastya.ryabova@nsc.gpi.ru (A.V.R.); 2 Koltzov Institute of Developmental Biology RAS, 119334 Moscow, Russia; t.tortunova@gmail.com (T.A.T.); kuzyaeva.valeriya@mail.ru (V.I.K.); 3 Moscow Center for Advanced Studies, 123592 Moscow, Russia; 4 Nanobiomedicine Division, Sirius University of Science and Technology, 354340 Sirius, Russia; 5 Shemyakin-Ovchinnikov Institute of Bioorganic Chemistry of the Russian Academy of Sciences, 117997 Moscow, Russia; griaznova.oly@gmail.com

**Keywords:** macrophages, biodistribution, magnetic nanoparticles, multimodal imaging, biodiagnostics, non-invasive monitoring, magnetic particle quantification

## Abstract

A variety of cells can be applied as vectors for the targeted delivery of chemotherapeutic or gene therapeutic agents to neoplasms. Macrophages are regarded as promising candidates for cell-based therapy. Accurate assessments of the efficacy and safety profiles of cell-based therapy products require the collection of data on their biodistribution and fate. The study of living cell distribution in vivo necessitates the utilization of a combination of methodologies to obtain more precise data regarding the fate of cells after their administration into animals. In the present study, a murine RAW 264.7 cell line was engineered to express enhanced green fluorescent protein (GFP). These cells were labeled with 50 nm magnetic nanoparticles (MNPs) for non-invasive real-time monitoring in mice using the magnetic particle quantification (MPQ) technique. The combination of high sensitivity and multimodality of the approach used permitted the acquisition of comprehensive data on the biodistribution of RAW-GFP cells in mice. For the first time, non-invasive, real-time monitoring of the dynamics of MNP-loaded macrophages in the bloodstream of mice has been achieved via the MPQ technique. Following intravenous administration, the cells are rapidly eliminated from the bloodstream, with subsequent accumulation mainly in the lungs and the liver. This may impose limitations on the use of such cells for drug delivery to other regions of a living organism.

## 1. Introduction

The potential of cell therapy approaches for the treatment of various diseases, including neoplastic [1,2,3,4], neurodegenerative [5,6,7], cardiovascular [8,9,10], and other conditions, is significant. Among the various cells investigated for cell therapy, macrophages have been identified as promising vehicles for the targeted delivery of chemotherapeutic and gene therapeutic agents [11,12,13]. This is due to the intrinsically high mobility and ability to undergo chemotaxis of these cells, as well as their easy penetration through peripheral tissues, including inflamed and neoplastic ones, and the high secretory activity that allows macrophages to modulate the microenvironmental inflammatory state [14,15,16]. Any cell type intended for use in cell therapy and/or drug delivery must undergo comprehensive biodistribution characterization during preclinical studies.

Due to their natural ability to perform phagocytosis, macrophages can be readily loaded by various exogenous labels, including fluorescent and magnetic ones. A plethora of options exists for such an approach, and the particles utilized can be nano- and micro-sized. Sapach and co-authors [17] employed microcapsules with Cy7 or RITC (rhodamine B isothiocyanate) to visualize macrophage-like cell lines and bone marrow-derived macrophages. The method enabled the detection of labeled macrophages both in vitro and in vivo for a duration of 24 h subsequent to the intravenous injection. Organic protein nanocontainers with encapsulated fluorescent protein appear to be promising candidates for both labeling [18] and drug delivery [19]. A two-color fluorescently tagged perfluorocarbon nanoemulsion (PFC-NE) was applied to track macrophages in diabetic neuropathy [20]. Fluorescent polymer dots have also been employed to track Ana-1 macrophages in deep organs and lymph nodes in mice [21]. Inorganic agents, such as gold nanoparticles, are actively phagocytosed by macrophages and have been used to study the interactions of tumor-associated macrophages (TAMs) with the tumor microenvironment [22]. Optical imaging methods have been demonstrated to be instrumental in the observation of the behavior and functional roles of macrophages in vitro. However, the application of these methods for in vivo studies is constrained by the low spatial resolution of 1–10 mm and the limited signal penetration depth of 1–2 cm, which is attributable to light scattering and absorption by tissues [23,24].

In the context of studies employing laboratory animals, superparamagnetic iron oxide nanoparticles (SPIONs) are frequently used due to their T2 contrast properties, which facilitate the observation of macrophage distribution through magnetic resonance imaging (MRI) analysis. For instance, SPIONs have been applied to target M2 macrophages and reverse their polarization to the M1 phenotype [25]. In vivo imaging of macrophage response to hepatic radiofrequency ablation [26] was achieved using SPIONs and gadolinium-160-based contrast agents. The indisputable benefit of magnetic labels is their high penetrating signal. Nevertheless, MRI does not provide data on the viability of the visualized cells.

The tracking of macrophages can also be accomplished using positron emission tomography (PET) imaging. In a particular study [27], mouse macrophages were labeled with aza-dibenzocyclooctyne-tethered PEGylated mesoporous silica nanoparticles with an F-18-labeled azide radiotracer (18F-DBCOT-MSNs). This enabled the detection of RAW 264.7 macrophages within tumor and atherosclerotic plaque sites in tumor-bearing and ApoE -/- mouse models. The authors of ref. [28] reported macrophage tracking in mouse models of cardiovascular disease and cancer with apolipoprotein-inspired radiotracers (an ApoA1-mimetic peptide radiolabeled with zirconium-89). In a study by Varasteh and co-authors, gallium-68-labeled nanobodies targeting the macrophage mannose receptor were applied to monitor macrophages within the myocardium after myocardial infarction [29]. The primary constraint imposed by both fluorescent and magnetic exogenous labels pertains to a decrease in signal intensity during the cell proliferation process. Nonetheless, it has been demonstrated that even rapidly dividing cells, such as malignant ones, can be detected for a period of at least five or seven days after the implantation [30,31].

A number of stable macrophage cell lines have been generated using lentiviral and adenoviral transduction [32,33]. It has also been reported that a primary culture of mouse macrophages containing both GFP and a bioluminescent reporter has been established [34].

In the present study, we have combined optical and magnetic techniques for the labeling of macrophages. Optical labeling was accomplished thought the use of the enhanced GFP, stably expressed in the RAW-GFP macrophage cells. This labeling facilitated the detection of the fluorescent signal of the administered cells in various tissues using confocal microscopy and optical tomography. As a complementary label, we employed 50 nm glucuronic acid-coated iron oxide magnetic nanoparticles (MNPs). The detection of the fluorescent label necessitated the preparation of tissue suspensions or histological sections. Conversely, MNP-labeled macrophages could be detected noninvasively in vivo in the bloodstream using the magnetic particle quantification (MPQ) technique, as well as ex vivo by the accumulation of intracellular iron.

The utilization of a dual-labeling strategy, integrated the two aforementioned approaches, has been demonstrated to facilitate real-time monitoring of the labeled macrophages in mice. Following intravenous administration, the macrophages are rapidly eliminated from the bloodstream and accumulate mainly in the liver and the lungs. In the context of potential applications of macrophages in cell-based therapy, the rapid clearance of cells from the bloodstream, together with their subsequent accumulation in the lungs and the liver, limits the use of such a delivery strategy for targets located elsewhere in the organism.

## 2. Results and Discussion

### 2.1. Establishment of the RAW-GFP Cell Line and MNPs Uptake by the Obtained Cells

**Figure 1:**
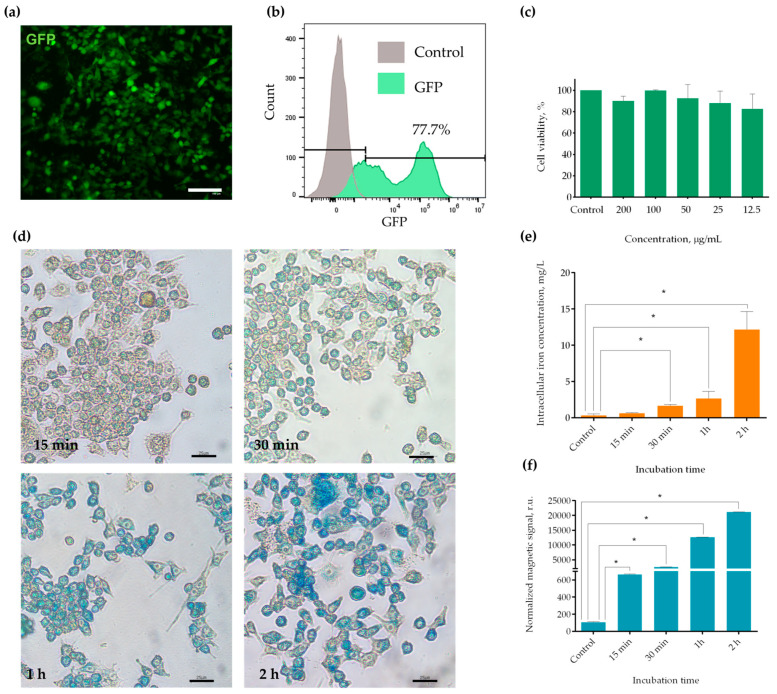
Characterization of the RAW-GFP cell line and MNP uptake by the obtained cells. GFP fluorescence in RAW-GFP macrophages assessed by (**a**) fluorescent microscopy (scale bar—100 µm) and (**b**) flow cytometry; (**c**) viability of the obtained cells in the presence of different concentrations of MNPs, evaluated by the resazurin assay; (**d**) Prussian blue staining of RAW-GFP cells after 15 min, 30 min, 1 h, and 2 h of incubation with MNPs, scale bars are 25 µm; (**e**) MNP accumulation in RAW-GFP macrophages quantified by ICP-MS; (**f**) dynamic assessment of MNP capture by RAW-GFP cells after 15 min, 30 min, 1 h, and 2 h of incubation, evaluated with the MPQ method. All data are shown as the mean + S.D. of three independent experiments; * indicates *p*-value < 0.001.

In the present study, an immortalized macrophage-like cell line was utilized, which was characterized by rapid proliferation yet possessed numerous characteristics of primary macrophages. In order to obtain a cell line with a stably expressed fluorescent label, a lentiviral vector was constructed. This vector carried genes that encode for GFP and puromycin resistance. Our data indicated that a short GFP transgene sequence could be integrated into the genome with a single round of transduction with subsequent selection. As illustrated in Figure 1a, a micrograph of the resulting RAW-GFP cells reveals a cytoplasmic localization of the bright green fluorescent signal. The number of GFP^+^ cells was assessed by flow cytometry (Figure 1b). It was demonstrated that approximately 78% of the obtained cells expressed GFP.

As previously stated, MNPs were used as a complementary label. We employed an aqueous solution of glucuronic acid-coated iron oxide MNPs (a representative TEM image of the MNPs is shown in Supplementary Figure S1). These MNPs have previously been comprehensively characterized, demonstrated good detectability by nonlinear magnetization using the MPQ technique, and have shown biocompatibility and safety in animal models [35,36,37]. In the present study, we aimed to evaluate MNP toxicity to the obtained cells, as well as to select an optimal MNP concentration for labeling cells before administration. The assessment of MNP toxicity was conducted within the concentration range from 12.5 μg/mL to 200 μg/mL, employing the resazurin test (Figure 1c, Supplementary Figure S2). The analysis revealed that MNPs did not exhibit toxic effects at any of the analyzed concentrations.

Subsequently, a dynamic assessment of MNP accumulation by macrophages was performed via an evaluation of iron accumulation in the cells using Perls staining (Figure 1d) and ICP-MS (inductively coupled plasma mass spectrometry, Figure 1e). Both methods indicated that the accumulation of nanoparticles occurred in a time-dependent manner. The cells exhibited rapid phagocytosis of MNPs within 15 min, with further accumulation over the course of 2 h. The amount of intracellular iron quantified by ICP-MS after 2 h of incubation of RAW-GFP cells with MNPs was 434 ± 0.9 fg per cell. A concentration of 200 μg/mL was selected for further studies, as it did not have pronounced toxicity (90 ± 4% of the cells were viable), concurrently enabling visualization of the cells in the Prussian blue reaction. The latter feature was important for validating the results in the histological study.

Given the superparamagnetic properties of the MNPs, the dynamics of their uptake by the cells were quantified by the MPQ method. For this purpose, the cells were exposed to MNPs for various durations (15 min, 30 min, 1 h, and 2 h). Thereafter, the unbound MNPs were eliminated from the cells through a series of centrifugation and washing steps. The cuvettes containing cell-bond nanoparticles were positioned inside the measuring coil of the MPQ device. The magnitude of the normalized magnetic signals (Figure 1f) was determined in cell pellet samples (3 × 10^6^ cells per sample).

The obtained results are in good agreement with the data acquired when assessing the iron amount in MNP-labeled RAW-GFP cells using ICP-MS and Perls staining.

### 2.2. Multimodal Detection of MNP-Labeled Macrophages In Vivo and Ex Vivo

**Figure 2:**
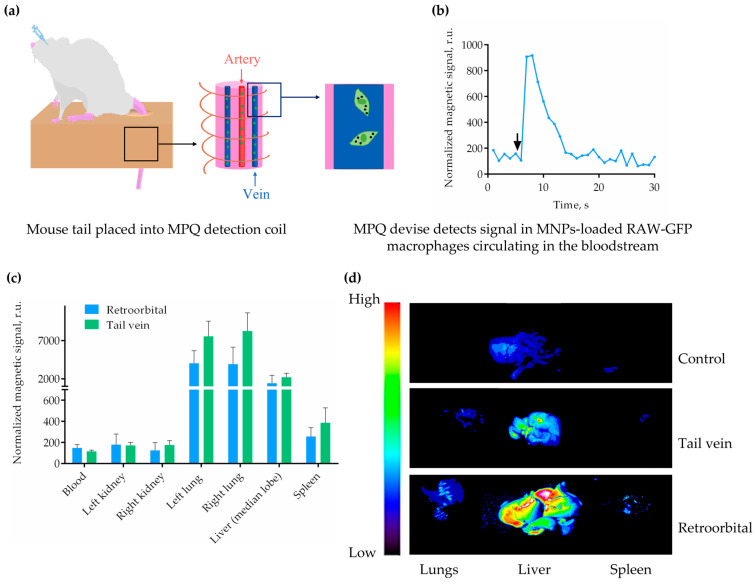
The distribution of MNP-loaded RAW-GFP cells in mice after intravenous administration. (**a**) Experimental setup: the tail was placed into the measuring coils of the MPQ device; the RAW-GFP cells pre-labeled with MNPs were injected into the retro-orbital venous sinus; and the magnetic signals were recorded in real time in the tail veins and arteries of mice. (**b**) Representative data from real-time magnetic signal measurements of cell circulation in the bloodstream; the arrow indicates the moment of cell infusion. (**c**) Magnetic signals measured in the organs of mice with the MPQ device; the data are presented as mean + S.D., *n* = 4 for each group. (**d**) Representative fluorescence images of the lungs, the liver, and the spleen in non-injected mice (upper panel) and MNP-loaded RAW-GFP cell-injected mice (“tail vein” and “retro-orbital” groups—middle and bottom panels, respectively). Ex vivo fluorescence images of whole organs were obtained using the LumoTrace bioimaging system; the spectrum gradient bar corresponds to the GFP fluorescence intensity; *n* = 3 for each group.

The subsequent task was to investigate the in vivo distribution of dual-labeled RAW-GFP cells after intravenous administration. Two commonly used routes of administration were selected for this study: the lateral tail vein and the retro-orbital venous sinus [38,39]. As previously stated, the MPQ device is capable of measuring the magnetic signal in both isolated organ samples and in the bloodstream, exhibiting a time resolution of approximately 1 s. For the first time, non-invasive real-time monitoring of the dynamics of MNP-labeled macrophages in the bloodstream of mice was realized via the MPQ technique. The experimental setup is illustrated in Figure 2a. The mouse tail was placed into the measuring coil of the MPQ device, and the signals were recorded in real time after injecting the cells retro-orbitally. It was ascertained that MNP-loaded RAW-GFP cells were rapidly cleared from the bloodstream (within 10–15 s, Figure 2b), which is approximately the one-pass circulation time of blood in BALB/c mice [40,41]. The MPQ technique has been demonstrated to offer significant advantages over conventional methods. Firstly, it eliminates the need for repeated blood sampling from laboratory animals, which are often used to determine the circulation time of cells or drugs in the blood [42,43]. Secondly, it provides a substantially higher level of sensitivity compared to MRI and intravital microscopy [44].

The distribution of MNP-labeled cells was examined in the kidneys, lungs, spleen, and liver, as well as in the blood collected 2 h after the administration of RAW-GFP cells. According to the MPQ measurements, the cells were predominantly detected in the lungs and the liver, and significantly weaker signals were also noted in the spleen. No statistically significant differences were revealed between the “tail vein” and “retro-orbital” groups (Figure 2c). It is known that retro-orbital venous sinus injection is the less stressful of the two techniques [45,46]; therefore, retro-orbital administration is recommended for future studies using macrophages as therapeutic agents or drug carriers.

An optical imaging system was employed to detect the fluorescent signal in organs exhibiting high magnetic signals, including the lungs, the liver, and the spleen (Figure 2d, Supplementary Figure S3). Despite the use of the GFP fluorophore, which, contrary to near-infrared labels, has a comparatively augmented absorption of light by tissues and a diminished depth of light penetration [47], the outcome was consistent with the data obtained by the MPQ device. The lungs and livers of mice after the administration of MNP-labeled RAW-GFP cells had a significantly higher fluorescent signal, compared to the organs of non-injected mice that were used as an autofluorescence control. As before, no significant differences were identified between the “tail vein” and “retro-orbital” groups.

The cells’ short circulation time and subsequent accumulation in the lungs and the liver may limit the ability of such cells to deliver drugs to targets located in other areas of the living organism. In order to enhance the capacity of the cells for drug delivery, it is necessary to develop advanced methods that can increase the circulation time of the cells in the bloodstream, similar to the principle realized for nanoparticles [48].

### 2.3. Histological Analysis of MNP-Loaded Cell Distribution

**Figure 3:**
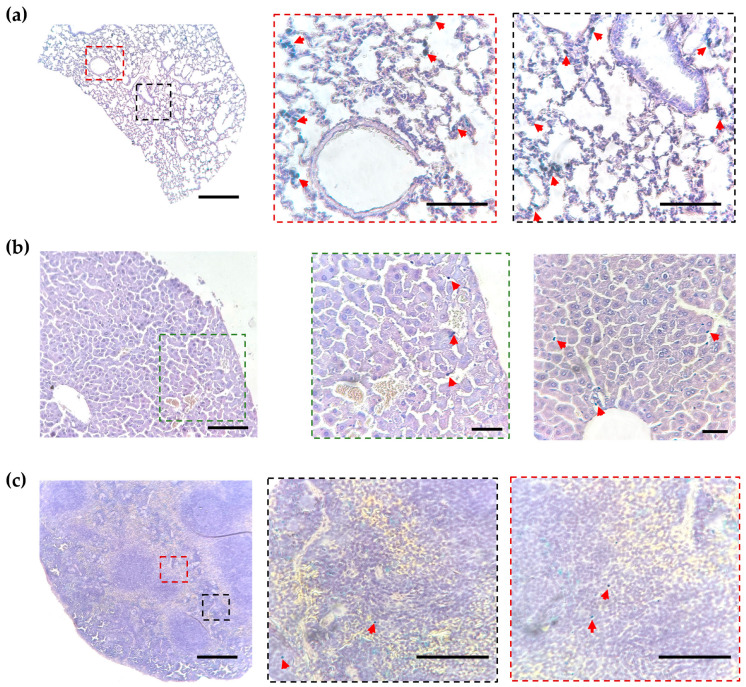
Representative histological images of the lung (**a**), liver (**b**), and spleen (**c**); hematoxylin–eosin and Prussian blue staining, where red arrows indicate iron deposits in RAW-GFP cells. Bright-field microscopy; scale bars are 500 µm and 50 µm (in the close-up images highlighted by dotted squares).

The detection of fluorescent signals in isolated organs may be complicated by several factors, including an uneven distribution of the administered cells in the tissue, the peculiarities of light propagation, and the presence of different autofluorescence signals in the organs. Therefore, a histological examination of the sections was conducted to facilitate a comparative analysis with the data obtained by the MPQ technique. Figure 3 and Supplementary Figure S4 provide representative histological images of the lungs, liver, spleen, and kidney, stained with hematoxylin–eosin and Prussian blue. Iron-positive cells were found throughout the lung tissue, with a predominant localization around large vessels (Figure 3a). In comparison with the lung, the liver did not reveal such extensive infiltration; however, cells were found near vessels and in the vascular lumen (Figure 3b). Significantly fewer cells were identified in the spleen pulp (Figure 3c). The iron-positive cells were practically undetected in the kidneys (Supplementary Figure S4). Thus, the data obtained by the MPQ method are in good agreement with the results of the histological study.

**Figure 4:**
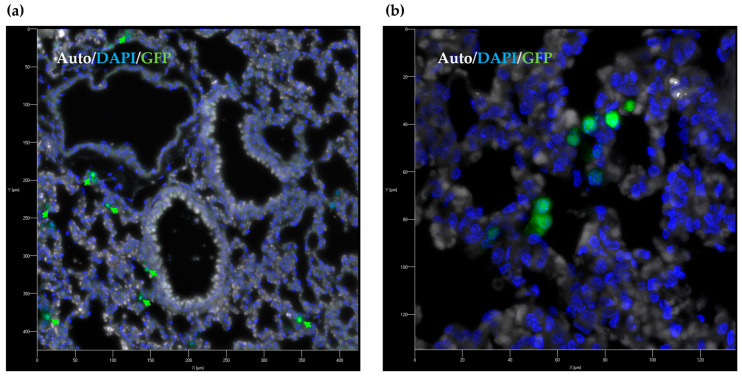
Distribution of MNP-labeled RAW-GFP macrophages in the lung, visualized by laser scanning confocal microscopy with maximum projection of the image volume; green arrows indicate RAW-GFP cells in the alveolar space. Green fluorescent signal—GFP; blue fluorescent signal—DAPI; gray color—autofluorescence signal; magnification 20× (**a**) and 63× (**b**).

Laser scanning confocal microscopy was employed to visualize MNP-labeled RAW-GFP cells in the lung histological sections (Figure 4, Supplementary Figure S5a,b). The cell distribution pattern revealed by confocal microscopy was shown to be similar to that obtained by Prussian blue staining; RAW-GFP cells were detected in the alveolar space and near vessels.

Representative confocal images of the kidney and the spleen are shown in Supplementary Figure S6a,b.

It should be mentioned that despite the extensive infiltration of the lung tissue by MNP-labeled RAW-GFP macrophages, no side effects such as dyspnea/respiratory arrest or any other signs of lung injury were observed in the animals throughout the study. To assess potential tissue damage in MNP-labeled RAW-GFP-enriched organs, a thorough analysis of all sections of the lung, liver, and spleen was conducted. The analysis revealed no evidence of tissue alterations, such as microhemorrhages or vascular occlusion by cells, in any of the sections examined.

Given that the accumulation of cells occurs mainly in the lungs, the resulting cells can be supplemented with a chemotherapeutic drug, or other transgenic sequences can be integrated into the cell genome to produce CAR (chimeric antigen receptor) macrophages that can be used for the therapy of pulmonary metastases. Conversely, given the short circulation time of the cells in the bloodstream after intravenous administration, it should be considered that the efficacy of such a delivery strategy may be limited in the treatment of malignant neoplasms of other localizations.

## 3. Materials and Methods

### 3.1. Materials

The following reagents were used: Roswell Park Memorial Institute (RPMI) 1640 medium, Dulbecco’s Modified Eagle Medium (DMEM), l-glutamine (Servicebio, Wuhan, China), OptiMEM (Minimum Essential Medium, Gibco, Waltham, MA, USA), Lipofectamine3000 (Thermo Fisher Scientific, Waltham, MA, USA), fetal bovine serum (FBS), penicillin/streptomycin, trypsin–EDTA (ethylenediaminetetraacetic acid, Servicebio, Wuhan, China), resazurin sodium salt (Thermo Fisher Scientific, Waltham, MA, USA), polybrene (Merck, Darmstadt, Germany), fluidMAG-ARA 50 nm glucuronic acid-coated magnetic nanoparticles (Chemicell, Berlin, Germany), formaldehyde, Mayer’s hematoxylin, eosin, hydrochloric acid, potassium hexacyanoferrate (II) trihydrate (Biovitrum, Moscow, Russia), and DAPI (4′,6-diamidino-2-phenylindole, Lumiprobe, Moscow, Russia).

### 3.2. Cell Lines and Culture

RAW 264.7 cells were cultured in RPMI-1640 medium supplemented with 5% FBS, 1% penicillin/streptomycin, and 2 mM l-glutamine at 5% CO_2_, 80% humidity, and 37 °C. The cells were passaged after reaching 80–90% confluence, detached with a cell scraper, and passaged in a 1:4–1:6 ratio to T-25 flasks. HEK293T cells were cultured in DMEM supplemented with 10% FBS, 1% penicillin/streptomycin, and 2 mM l-glutamine. For passaging, HEK293T cells were treated with 0.25% trypsin–EDTA and transferred to T-25 flasks at a 1:4–1:8 ratio.

### 3.3. Lentiviral Vector Construction and the Generation of GFP-Expressing RAW 264.7 Cells

Lentiviral vectors were assembled using the HEK293T packaging cell line. The cells were seeded into 6-well plates and cultured in full DMEM at 37 °C and 5% CO_2_. After 24 h of culturing, lentiviral packaging plasmids (pRSV-Rev, pMDLg/pRRE, and pCMV-VSV-G, Addgene, Watertown, MA, USA) and a plasmid carrying GFP encoding genes (LeGO-G/Puro [49]) were mixed with P3000 reagent in Opti-MEM medium. Diluted DNA was then added to diluted Lipofectamin3000 reagent (in a 1:1 ratio), and after 15 min of incubation, the resulting DNA–lipid complexes were added to the cells in 6-well plates. The next day, the medium was aspirated and replaced with the full DMEM growth medium. Lentiviral stocks were collected at 48 and 72 h after co-transfection and filtered through a 0.45-μm syringe filter.

The transduction of RAW 264.7 cells with a lentiviral vector was performed using a standard protocol. Briefly, the cells were cultured in RPMI-1640 medium, supplemented with 10% heat-inactivated FBS, 2 mM l-glutamine, and 8 μg/mL polybrene; 48 h after the transduction, selection was carried out using puromycin at a concentration of 500 ng/mL; the medium with puromycin was replaced with a new one every other day. The resulting cell line was named RAW-GFP.

### 3.4. Fluorescent Microscopy

Fluorescent micrographs of RAW-GFP cells were captured with the FLoid Cell Imaging Station (Thermo Fisher Scientific, Waltham, MA, USA).

### 3.5. Flow Cytometry

GFP expression in RAW-GFP cells was assessed using a CytoFLEX S Flow Cytometer (BD Biosciences, Franklin Lakes, NJ, USA) and the CytEXPERT v. 2.4.0.28 software (Beckman Coulter, Brea, CA, USA). The results were analyzed using the FlowJo v. 10.8.1 software (TreeStar, BD Bioscience, Franklin Lakes, NJ, USA).

### 3.6. Cytotoxicity Assay

The impact of fluidMAG-ARA MNPs on the viability of RAW-GFP cells was evaluated using the resazurin assay according to the manufacturer’s instructions. The RAW-GFP cells were seeded into the wells of a 96-well opaque culture plate at a density of 1 × 10^5^ cells per well in 150 μL of a growth medium. Following 24 h of culture, MNPs were added to the cells at different concentrations (12.5, 25, 50, 100, and 200 µg/mL). After 24 h, 48 h, and 72 h of incubation, the cells were washed with PBS, and a fresh growth medium containing 50 μM of resazurin sodium salt was added to each well. The cells were incubated with resazurin for 4 h at 37 °C and 5% CO_2_ within a humidified atmosphere. The analysis was performed in triplicate, and the intensity of the fluorescent signal was quantified using a Feyond A400 microplate reader (Hangzhou Allsheng Instruments, Hangzhou, China) at λ_em_ = 560 nm. The number of metabolically active cells was assessed using the following formula:

N = (A_s_−A_b_ / A_c_−A_b_) × 100%

where A_s_ is the average value of fluorescence intensity in sample wells, A_b_ is the average value of fluorescence intensity in blank wells (no cells seeded), and A_c_ is the average value of fluorescence intensity in control wells (no MNPs added).

### 3.7. Prussian Blue Staining of Cells and Tissue Sections

After 15 min, 30 min, 1 h, and 2 h of incubation of RAW-GFP cells with MNPs at a concentration of 200 µg/mL, the cells were washed with PBS and fixed with 4% paraformaldehyde in PBS. Subsequently, the cells were washed with deionized water and stained using an Iron Staining Kit (Biovitrum, Moscow, Russia). Afterwards, the cells were washed again with deionized water.

Paraffin-embedded 5 µm sections of lungs, liver, kidneys, and spleen were stained by Prussian blue for detecting the MNP-labeled RAW-GFP cells according to the protocol described above. Images were taken using a CX41 FLLED light microscope (Ningbo Sunny Instruments, Yuyao City, China).

### 3.8. Inductively Coupled Plasma Mass Spectrometry (ICP-MS)

After 15 min, 30 min, 1 h, and 2 h of incubation of RAW-GFP cells with MNPs (200 µg/mL), the cells were thoroughly washed with PBS, detached from the plastic using the trypsin solution, and pelleted by centrifugation before counting. Each cell sample (3 × 10^6^ cells) was dissolved in 100 µL of concentrated nitric acid (2 h at 60 °C). The concentration of iron was then determined with a NexION 2000 Inductively Coupled Plasma Mass Spectrometer (PerkinElmer, Waltham, MA, USA).

### 3.9. Magnetic Particle Quantification (MPQ)

Real-time detection of magnetic nanoparticles in cell pellets, isolated organs, and non-invasively in the bloodstream was carried out using an updated device with a high temporal resolution of up to 1 s based on the magnetic particle quantification (MPQ) method [50,51]. The MPQ employs a nonlinear magnetization of ferromagnetic and superparamagnetic MNPs were subjected to a magnetic field at frequencies *f*_1_ = 80 Hz and *f*_2_ = 80 kHz, with detection of the response at the combinatorial frequency *f*_2_ + 2*f*_1_.

### 3.10. Animals

Six- to eight-week-old female BALB/c mice (15–25 g) were obtained from the Stolbovaya animal facility, a branch of the Scientific Center for Biomedical Technologies of the Federal Medical and Biological Agency of the RF. The mice were housed in a temperature-controlled facility under a 12 h photoperiod, no more than ten per cage. The mice were given food and water ad libitum.

### 3.11. Histological Study

After 2 h of incubation of RAW-GFP cells with MNPs (200 µg/mL), the cells were washed with PBS, detached with a cell scraper, counted, and injected into the tail vein or the retro-orbital venous sinus of mice (3 × 10^6^ cells in 350 μL of serum-free RPMI). For histological analysis, the mice were euthanized by cervical dislocation 2 h after the injection of MNP-labeled RAW-GFP cells. Lungs, livers, kidneys, and spleens were extracted, fixed in 10% formaldehyde for 24 h, embedded in paraffin, sectioned at a thickness of 5 μm, and stained with H-E and Prussian blue.

### 3.12. Optical Tomography

Fluorescent images of mouse organs extracted 2 hours after injection of RAW-GFP cells were received via LumoTrace FLUO bioimaging system (Abisense, Sirius, Russia) using 450 and 470 nm diodes and a 550+ nm filter with an exposure time of 100 ms.

### 3.13. Laser Scanning Confocal Microscopy

Fluorescence microscopy analysis of the paraffin-embedded tissue sections was performed using an LSM-710 laser scanning confocal microscope (Carl Zeiss Microscopy, Oberkochen, Germany), with a 20× Plan-Apochromat objective (NA = 0.8). The linear separation mode was implemented by recording fluorescence with a 32-channel GaAsP detector and separating that signal into contributions from the GFP (green channel) and autofluorescence (gray channel) spectra, pre-registered in the spectral range of 500–650 nm with excitation by a laser at a wavelength of 488 nm. The DAPI fluorescence was excited at 720 nm (two-photon excitation), using a femtosecond laser (Chameleon Ultra II, Coherent, USA), and was detected in the range from 400 to 500 nm. The 3D fluorescence images were acquired with a step of 1 µm along the Z-axis and reconstructed using the ZEN v. 1.1.2.0 software (Carl Zeiss Microscopy, Oberkochen, Germany). As a result, an overlay of GFP fluorescence, autofluorescence, and DAPI fluorescence 3D images was obtained.

### 3.14. Statistical Analysis

All experiments were performed at least in triplicate. The statistical analysis was performed using the Prism v.6 software (GraphPad, Boston, MA, USA). The significance of the differences was evaluated using one-way ANOVA followed by Dunnett’s post hoc test. Where relevant, a two-tailed unpaired *t*-test was used to determine the differences between the treated cells and the control; a *p*-value < 0.05 was considered as significant. The results are presented as means + standard deviations.

## 4. Conclusions

In this study, murine RAW macrophages were stably modified with GFP-encoding genes and additionally labeled with 50 nm MNPs before intravenous and retro-orbital venous sinus administration to mice. The internalization of MNPs in the obtained cells was dynamically assessed after 15 min, 30 min, 1 h, and 2 h of incubation with RAW-GFP cells by the MPQ technique, ICP-MS, and Perls reaction. It has been demonstrated that the accumulation of MNPs in the cells occurs in a time-dependent manner. The toxicity of MNPs was assessed through the implementation of the resazurin assay, which revealed no pronounced toxic effect. The quantity of MNP-loaded macrophage cells in the circulation was recorded in real time via the MPQ technique to provide non-invasive monitoring of the distribution dynamics of the MNP-loaded RAW-GFP cells in mice. It has been found that the cells rapidly clear from the bloodstream (within 10–15 s), with subsequent accumulation mainly in the lungs and the liver. The data obtained with the MPQ device were corroborated by histological examination of organ sections that had been stained with Prussian blue. The iron-positive cells were predominantly detected in the lungs and the liver. The distribution pattern of cells observed through confocal microscopy exhibited a high degree of similarity to that detected by Prussian blue staining. In order to enhance the efficacy of drug delivery by cells to other organs, it is imperative to explore advanced methodologies for prolonging the circulation time of these carriers.

## Data Availability

Data are contained within the article and Supplementary Materials.

## Supplementary Materials

The following supporting information can be downloaded at https://www.mdpi.com/article/10.3390/molecules30183726/s1, Figure S1: TEM image of FluidMAG-ARA MNPs. Figure S2: Viability of the obtained cells in the presence of different concentrations of MNPs, evaluated by the resazurin assay. Figure S3: Fluorescent images of extracted organs of non-injected mouse and MNPs-loaded RAW-GFP cells injected mouse. Figure S4: Representative histological images of kidney stained with hematoxylin-eosin and Prussian blue. Figure S5: Distribution of RAW-GFP macrophages in lung visualized by laser scanning confocal microscopy. Figure S6: Representative confocal images of kidney and spleen obtained by laser scanning confocal microscopy.
