# Supplementary material for: Multimodal Detection of Magnetically and Fluorescently Dual-Labeled Murine Macrophages After Intravenous Administration
Source: Molecules. 2025 Sep 12;30(18):3726. doi: 10.3390/molecules30183726 (PMC12472669; doi:10.3390/molecules30183726)
Supplement: Supplementary file 1 [file molecules-30-03726-s001.zip › molecules-3801610-supplementary.pdf]

Supplementary materials: Multimodal Detection of Magnetically and Fluorescently Dual-Labeled Murine Macrophages after Intravenous Administration

Anna N. Gabashvili, Sergey L. Znoyko, Anastasia V. Ryabova, Elizaveta N. Mochalova, Olga Yu. Griaznova, Tatiana A. Tortunova, Olga N. Sheveleva, Nina N. Butorina, Valeriia I. Kuziaeva , Irina V. Lyadova, Petr I. Nikitin

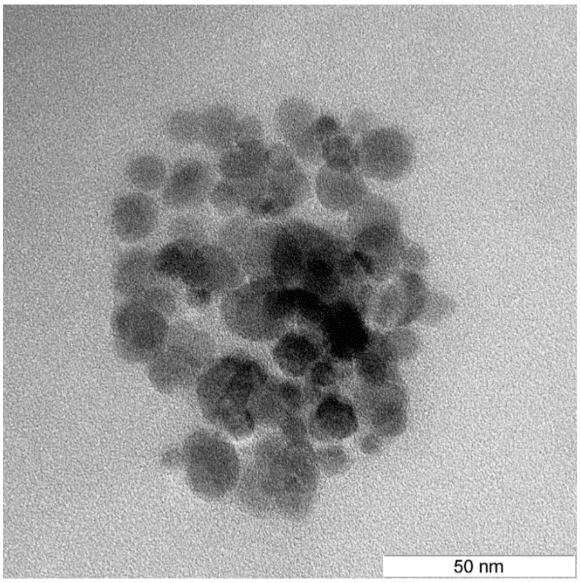

Supplementary Figure S1. TEM image of FluidMAG-ARA MNPs, scale bar – 50 nm.

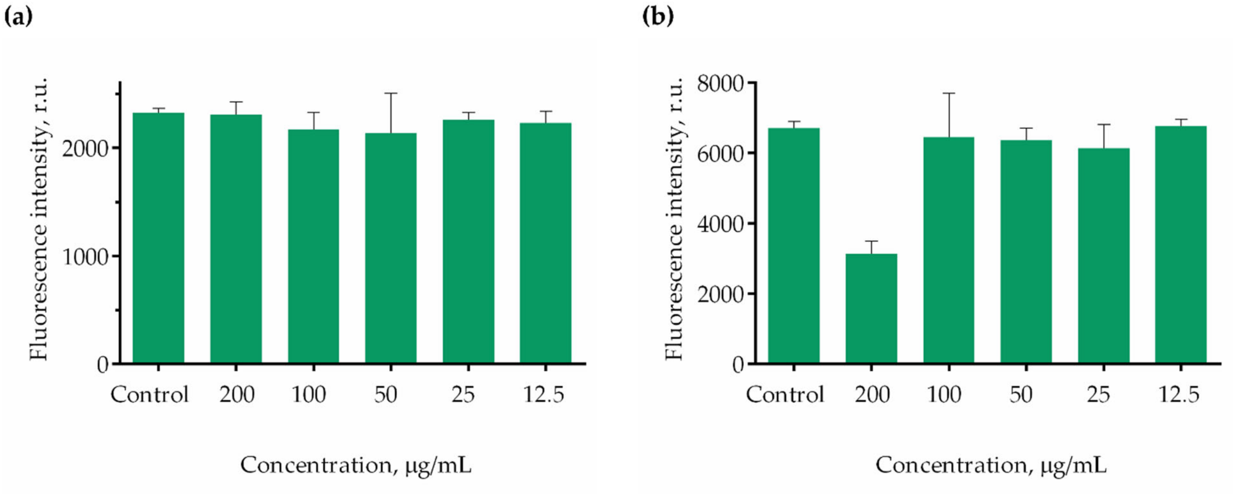

Supplementary Figure S2. Viability of the obtained cells in the presence of different concentrations of MNPs, evaluated by the resazurin assay after 48 h **(a)** and 72 h **(b)** of incubation of RAW-GFP cells with MNPs.

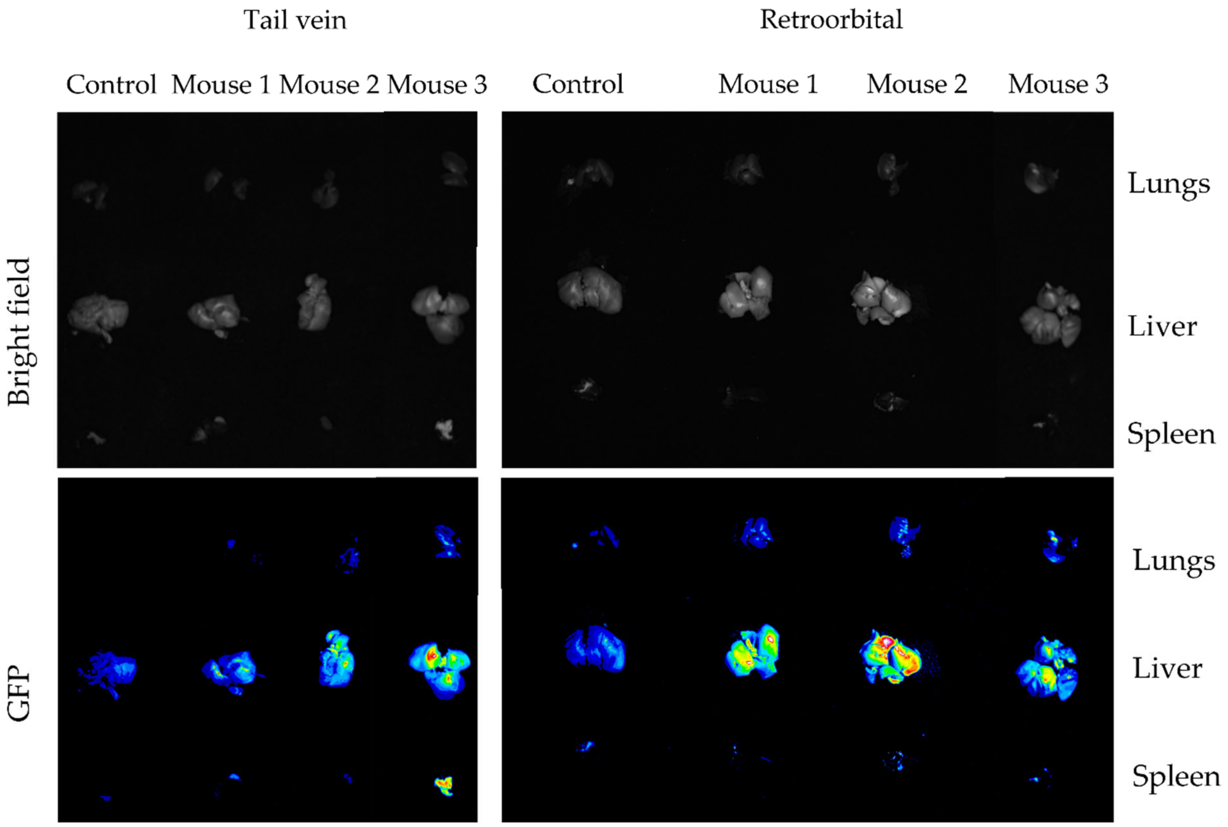

Supplementary Figure S3. Fluorescent images of extracted organs of non-injected mouse (Contol) and MNPs-loaded RAW-GFP cells injected mouse ("tail vein" and "retro-orbital" groups) obtained using the LumoTrace bioimaging system (λex = 450 and 470 nm, λem = 550+ nm).

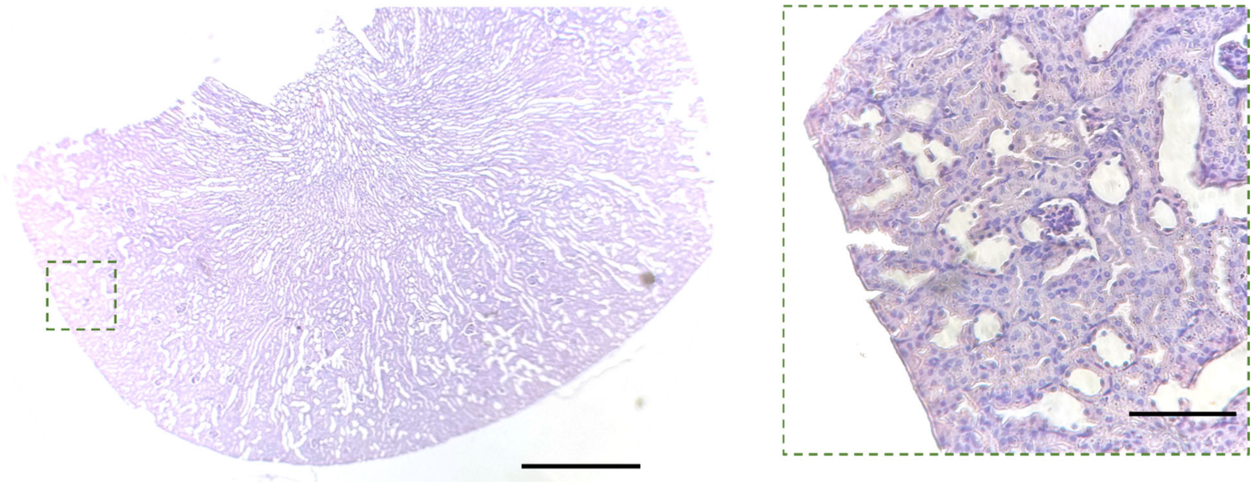

Supplementary Figure S4. Representative histological images of kidney stained with hematoxylin-eosin and Prussian blue. Bright-field microscopy, scale bars are 500 μm and 50 μm (in the close-up image highlighted by dotted square)

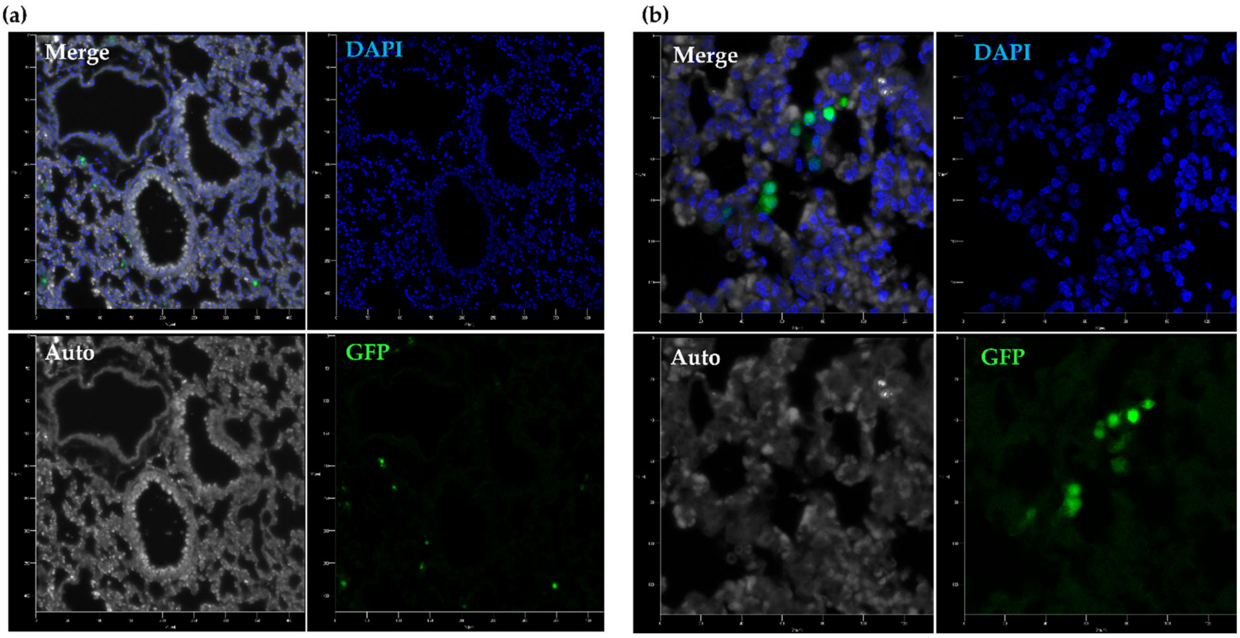

Supplementary Figure S5. Distribution of RAW-GFP macrophages in lung visualized by laser scanning confocal microscopy, maximum projection of an image volume; green fluorescent signal – GFP, blue fluorescent signal – DAPI, grey color – autofluorescence signal; magnification 20× **(a)** and 63× **(b)**.

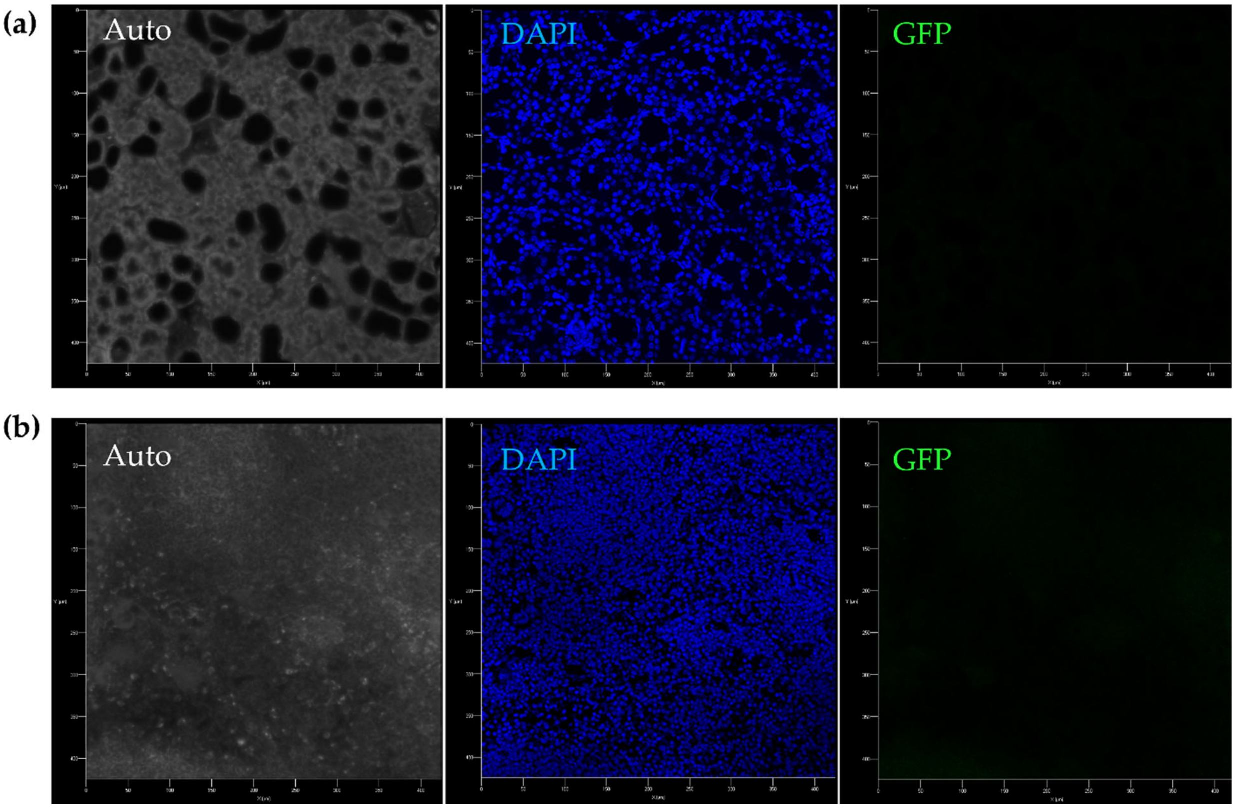

Supplementary Figure S6. Representative confocal images of kidney **(a)** and spleen **(b)** obtained by laser scanning confocal microscopy, maximum projection of an image volume; green fluorescent signal – GFP, blue fluorescent signal – DAPI, grey color – autofluorescence signal; magnification 20×
